# Graft Pre-conditioning by Peri-Operative Perfusion of Kidney Allografts With Rabbit Anti-human T-lymphocyte Globulin Results in Improved Kidney Graft Function in the Early Post-transplantation Period—a Prospective, Randomized Placebo-Controlled Trial

**DOI:** 10.3389/fimmu.2018.01911

**Published:** 2018-08-24

**Authors:** Paul V. Ritschl, Julia Günther, Lena Hofhansel, Anja A. Kühl, Arne Sattler, Stefanie Ernst, Frank Friedersdorff, Susanne Ebner, Sascha Weiss, Claudia Bösmüller, Annemarie Weissenbacher, Rupert Oberhuber, Benno Cardini, Robert Öllinger, Stefan Schneeberger, Matthias Biebl, Christian Denecke, Christian Margreiter, Thomas Resch, Felix Aigner, Manuel Maglione, Johann Pratschke, Katja Kotsch

**Affiliations:** ^1^Department of Surgery, Charité-Universitätsmedizin Berlin, Berlin, Germany; ^2^BIH Charité Clinical Scientist Program, Berlin Institute of Health, Berlin, Germany; ^3^Department of Visceral, Center for Operative Medicine, Transplant and Thoracic Surgery, Medical University of Innsbruck, Innsbruck, Austria; ^4^iPATH.Berlin—Immunopathology for Experimental Models, Charité-Universitätsmedizin Berlin, Corporate Member of Freie Universität Berlin, Humboldt-Universität zu Berlin, and Berlin Institute of Health, Berlin, Germany; ^5^Biostatistics Unit, Clinical Research Unit, Berlin Institute of Health, Charité-Universitätsmedizin Berlin, Berlin, Germany; ^6^Department of Urology, Charité-Universitätsmedizin Berlin, Berlin, Germany

**Keywords:** kidney transplantation, organ preservation, ATLG, ischemia reperfusion injury, RCT

## Abstract

**Introduction:** Although prone to a higher degree of ischemia reperfusion injury (IRI), the use of extended criteria donor (ECD) organs has become reality in transplantation. We therefore postulated that peri-operative perfusion of renal transplants with anti-human T-lymphocyte globulin (ATLG) ameliorates IRI and results in improved graft function.

**Methods:** We performed a randomized, single-blinded, placebo-controlled trial involving 50 kidneys (KTx). Prior to implantation organs were perfused and incubated with ATLG (AP) (*n* = 24 kidney). Control organs (CP) were perfused with saline only (*n* = 26 kidney). Primary endpoint was defined as graft function reflected by serum creatinine at day 7 post transplantation (post-tx).

**Results:** AP-KTx recipients illustrated significantly better graft function at day 7 post-tx as reflected by lower creatinine levels, whereas no treatment effect was observed after 12 months surveillance. During the early hospitalization phase, 16 of the 26 CP-KTx patients required dialysis during the first 7 days post-tx, whereas only 10 of the 24 AP-KTx patients underwent dialysis. No treatment-specific differences were detected for various lymphocytes subsets in the peripheral blood of patients. Additionally, mRNA analysis of 0-h biopsies post incubation with ATLG revealed no changes of intragraft inflammatory expression patterns between AP and CP organs.

**Conclusion:** We here present the first clinical study on peri-operative organ perfusion with ATLG illustrating improved graft function in the early period post kidney transplantation.

**Clinical Trial Registration:** www.ClinicalTrials.gov, NCT03377283

## Introduction

The growing demand for solid organs parallel with the decreasing number of potential donors has led to the use of organs derived from extended criteria donors (ECD) or organs retrieved after circulatory death (DCD) (1). However, these organs show a higher risk for unfavorable outcome as they are more susceptible to ischemia reperfusion injury (IRI) (2, 3). In the need to improve organ viability and function, most notably machine perfusion has been proven to exert beneficial effects on organ preservation in a number of experimental and clinical trials (4–7). However, alternative approaches for the conditioning of donor organs are missing so far.

IRI causes activation of the endothelium, resulting in increased permeability and increased expression of adhesion molecules, ultimately leading to increased attachment of inflammatory leukocytes to the endothelium (8). Moreover, reactive oxygen species, cytokines, chemokines, and adhesion molecules are secreted and released, augmenting the inflammatory immune response (9). Rabbit anti-thymocyte and anti-lymphocyte globulins (rATG/ATLG) have been reported to ameliorate IRI in numerous preclinical trials (10–12), primarily mediated by binding to E-selectin and ICAM-1 expressed by endothelial cells (13). Moreover, it was demonstrated that ATLG binds to activated endothelial cell *in vitro* (14). These data suggest that rATG/ATLG results in decreased inflammation and overall protection of the perfused tissue. Whereas rATG is the purified IgG fraction of sera from rabbits which have been immunized with human thymocytes, ATLG is a based on a preparation generated from rabbits immunized with the cultured T-acute lymphoblastic leukemia cell line Jurkat (15). In solid organ transplantation (SOT) rATG/ATLG is routinely applied in the prevention and treatment of solid organ graft rejection (16), mostly used as induction therapy (17, 18). Over the decades of its application it has been demonstrated that this drug is safe and highly potent in preserving allograft function following kidney transplantation (KTx) (19, 20). This is supported by the fact that the use of induction agents has increased over recent years as reflected by the finding that in 2013 more than 89% of kidney transplants in the United States were performed with induction therapy (21). As we and others have already illustrated that rATG induction therapy is also a safe and reasonable approach in liver transplantation (22, 23), we explored the potential for perfusing donor kidneys peri-operatively with ATLG in order to ameliorate IRI in a prospective, single-center, single-blinded, randomized placebo-controlled trial.

## Methods

### Patients

We performed a single-blinded, parallel randomized, placebo-controlled trial at the Department of Visceral, Transplant, and Thoracic Surgery of the Medical University of Innsbruck, Austria. An interdisciplinary transplantation board independently indicated the necessity for transplantation. Only adult patients receiving a kidney transplant from deceased donors were assessed for eligibility. Recipients diagnosed with HCV/HIV, undergoing re-transplantation or under a public guardian were not included.

### Study design

**Figure 1:**
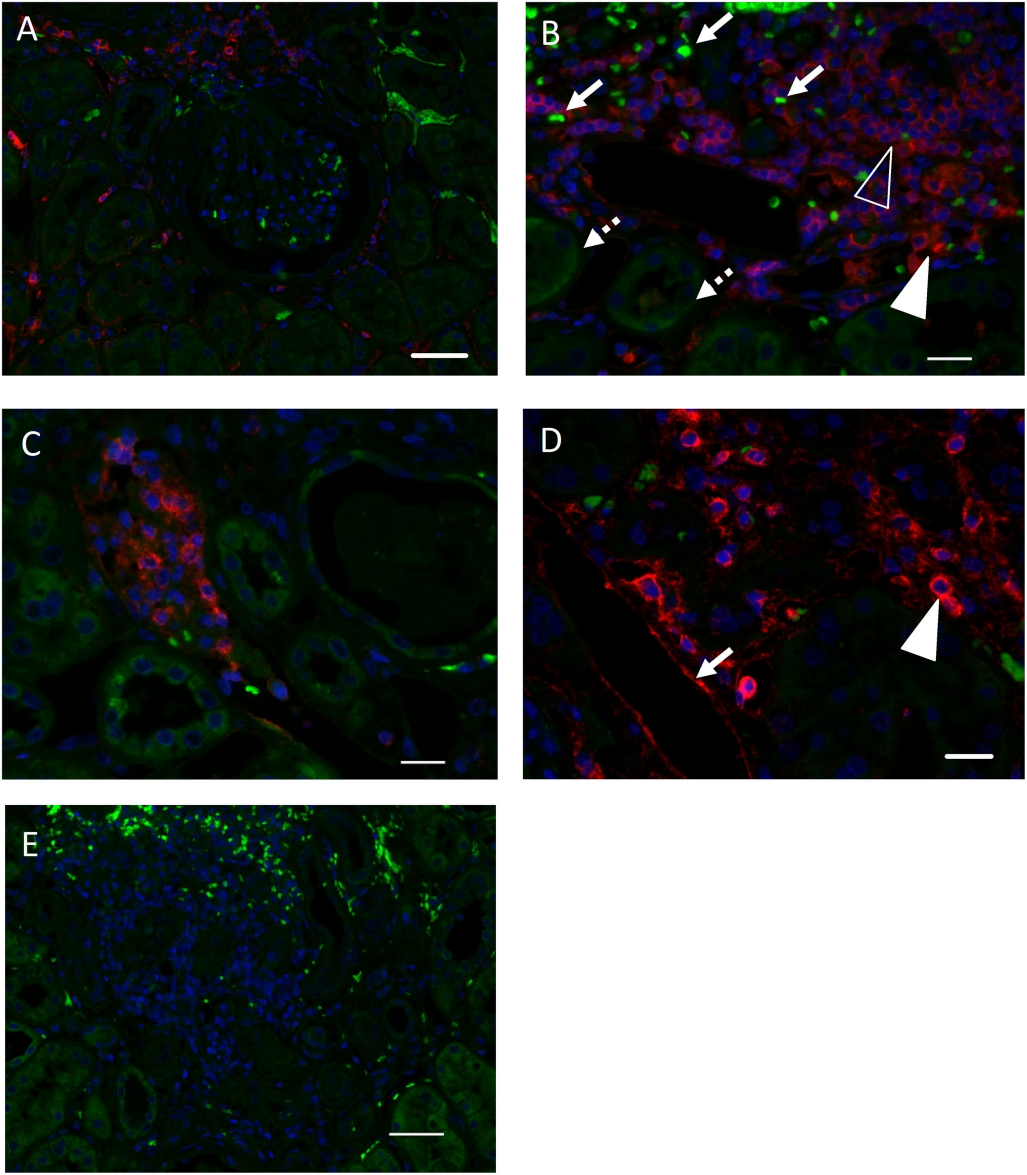
ATLG binding within the kidney. **(A)** ATLG binding to kidney structures was detected via binding of anti-rabbit polymer and visualization by Opal570 (red). Autofluorescence of the tissue (green) was kept in order to visualize kidney tissue morphology. ATLG shows no binding to structures within the glomerulus. Original magnification ×200, scale bar = 50 μm. **(B)** ATLG binds to intravascular and extravasated leukocytes within the kidney. Leukocytes within vessels (filled arrowhead) as well as leukocytes migrated into the kidney (blank arrowhead) show binding of ATLG (red). Autofluorescence of the tissue (green) was kept in order to distinguish tissue structures and to visualize erythrocytes (arrow) and tubules (dotted arrow). Original magnification ×400, scale bar = 20 μm. **(C)** Abundant leukocytes within a peritubular capillary show ATLG binding (red). Autofluorescence (green) helps to distinguish erythrocytes and tubules. Original magnification ×400. **(D)** ATLG (red) binds to the wall of peritubular capillaries (arrow) and leukocytes (arrowheads) within the kidney. Original magnification ×400, scale bar = 20 μm. **(E)** No binding of ATLG was detected in saline perfused control kidneys. Picture was taken with the same exposure time as used for treated kidney tissue sections. Original magnification ×200, scale bar = 50 μm. Representative pictures of *n* = 2 experiments with different organs are shown.

From 2012 to 2015 a total of 151 KTx recipients were assessed for eligibility (Figure 1). In total, 50 kidney recipients were eligible and enrolled in the study. Patients were randomly assigned to the treatment groups in a 1:1 ratio using block randomization and prepared randomization envelopes. For perfusion of kidneys, 12.5 mg of ATLG (Grafalon®, Neovii Biotech GmbH, Gräfelfing, Germany) was dissolved in 500 ml saline and administered via the renal artery. Organ perfusion was performed after back table preparation prior to implantation. After 5–10 min of incubation, vessels were flushed with HTK (Custodiol®, Dr. Franz Köhler Chemie GmbH, Deutschland) preservation solution to minimize systemic effects of “left-over” ATLG. Biopsies were taken 1 h after reperfusion. In addition, pre- (day−1) as well as post-transplantation (POD 0, 1, 3, 5, 7, 10, 15, month 3, and month 6), blood samples from recipients were collected, immediately processed and stored in liquid nitrogen until analysis. Immunosuppression was applied according to the center's protocol. Kidney recipients received basiliximab induction therapy and maintenance immunosuppression with tacrolimus, methylprednisolone, and mycophenolate mofetil (MMF). Observation period of the study was defined until month 12 post-transplantation (last patient visit).

### Endpoints

Primary endpoints of the study were determined as change in graft function at day 7 from baseline defined by serum. Key secondary endpoints were graft function and patient survival at the end of the follow-up period (12 months). Graft function was further assessed via estimated glomerular filtration rate (eGFR), and serum urea for KTx. eGFR values were calculated by means of the Modification of Diet in Renal Disease (MDRD4) formula (24). Further secondary endpoints were defined for functional parameters for time points other than primary outcome, frequency of acute rejection, cytokine and lymphocyte expression pattern. Clinical variables including recipient and donor age, donor body mass index (BMI), recipient age, recipient BMI, recipient sex, cold ischemia time (CIT), warm ischemia time (WIT), presence of panel-reactive antibodies (PRA), hospital stay, intensive care unit (ICU) stay, death/graft loss, number of HLA mismatches, and development of delayed graft function were considered for analysis.

### Flow cytometry

Mononuclear cells (MNC) from peripheral blood were isolated by Ficoll (Sigma-Aldrich Corp., St. Louis, MO, USA) density gradient centrifugation. For flow cytometry, 1 × 10^6^ cells were incubated for 15 min at 4°C with combinations of the antibodies listed in Supplemental Table 1. Data were analyzed using FlowJo software 9.3 (Tree Star Inc., Ashland, OR, USA), and the gating strategy is illustrated in Supplemental Figure 1A. Absolute leukocytes counts were evaluated at the Central Institute for Medical and Chemical Laboratory Diagnostics of the Innsbruck Medical University in a daily routine (Supplemental Figure 1B).

### Real-time RT-PCR

Real-time RT-PCR was performed as recently described (25). In brief, total RNA from snap-frozen biopsies was extracted using the NucleoSpin RNA Kit (Macherey-Nagel, Düren, Germany) according to the manufacturer's instructions. Integrity of RNA was checked using a NanoDrop™ 2000c spectrophotometer. Real-time reverse transcription polymerase chain reaction (RT-PCR) for gene expression analysis was performed with the ABI PRISM 7500 Sequence Detection System (Life Technologies, Carlsbad, CA, USA). Primers were directly purchased as Taqman® gene expression assays (Life Technologies, Carlsbad, CA, USA) (Supplemental Table 2). Specific gene expression was normalized to the housekeeping gene hypoxanthine-guanine phosphoribosyltransferase (HPRT) using the formula 2^−Δ*Ct*^.

### Histology

Kidney tissue samples were fixed in 4% formalin overnight and embedded in paraffin. Paraffin sections of 1–2 μm thickness were cut and dewaxed prior to heat-induced epitope retrieval. Endogenous peroxidase was blocked and sections incubated with an anti-rabbit polymer (EnVision; Agilent Santa Clara, CA, USA) followed by Opal570 (PerkinElmer, Hamburg, Germany). Sections were pressure cooked and nuclei stained with DAPI (Sigma-Aldrich, Darmstadt, Germany). After coverslipping with Fluoromount-G (Invitrogen GmbH, Karlsruhe, Germany) sections were analyzed using a Zeiss AxioImager Z1 (Carl Zeiss Microscopy GmbH, Jena, Germany). Controls were performed by omitting the anti-rabbit polymer.

### Statistics

Primary analysis was performed in the intention-to-treat (ITT) population. Missing data for the primary analysis of longitudinal clinical parameters (percentage of change from baseline prior to transplantation) were not imputed since mixed models were applied for comparison adjusted for baseline. Kaplan-Meier plots were used to analyze overall survival, and a log-rank test was applied to assess the statistical significance of differences between survival curves. Sensitivity adjustments were performed for age, recipient BMI, recipient gender. Due to simple or co-primary defined endpoints multiplicity adjustment was not required. Variables and endpoints were analyzed using appropriate parametric or non-parametric statistical methods based on their scale and distribution. All *p*-values from secondary analyses were considered in a non-confirmatory, exploratory way. Data were tested for normal distribution via visual analysis using histograms. Flow cytometry and PCR data were analyzed by applying a paired Mann-Whitney U test or Kruskal-Wallis (with Dunn *post-hoc*) test. A p value below 0.05 was considered statistically significant. Data analysis was performed using the statistical software SPSS (IBM V22.0) and GraphPad Prism 5.

### Study approval

This study was conducted according to the Declaration of Helsinki in accordance with good clinical practice guidelines, and approved by an independent institutional ethics committee of Innsbruck Medical University (ID: UN4640). All participants provided written informed consent.

## Results

### ATLG binding within the kidney

For *in situ* detection of ATLG binding, we determined binding of ATLG after perfusion of resected kidneys (*n* = 2) applying the same set-up as for the renal transplants. As displayed in Figures 1A–D, ATLG binds within a short incubation time of 5–10 min. to intravascular and extravasated leukocytes within the kidney as well as to the wall of peritubular capillaries. No binding of ATLG was detected in saline perfused control kidneys.

### Patients

**Figure 2:**
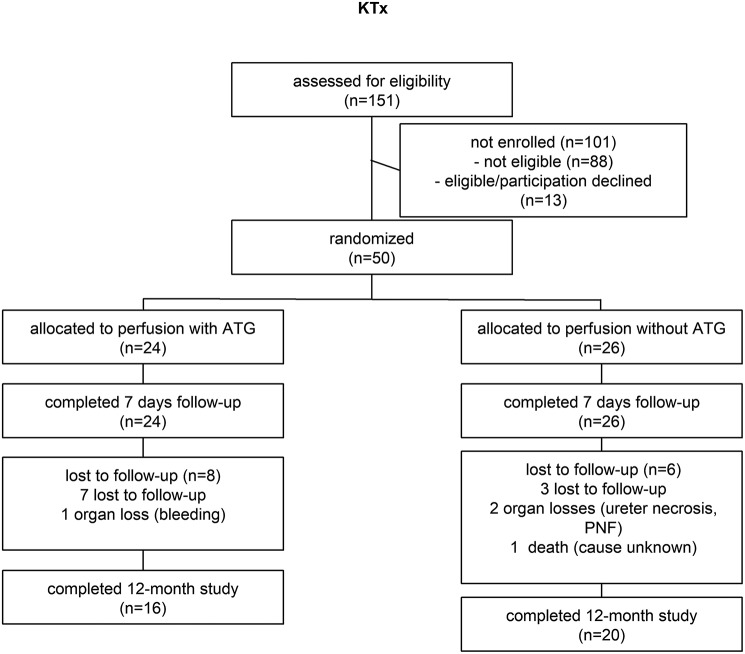
ATLG kidney perfusion consort diagram.

In total, 24 subjects were randomly allocated to receive a kidney transplant perfused with ATLG and 26 patients were randomly allocated to receive a kidney transplant perfused with saline only. Baseline characteristics were similar between both groups (Table 1). In the ATLG group *n* = 8 patients and in the control group *n* = 6 patients were lost to follow-up. After 12 months *n* = 16 (ATLG) and *n* = 20 (control) patients completed the observation period (Figure 2). No differences were detected for donor and recipient age, gender or ischemic times. Whereas only ten of the 24 patients in the ATLG group developed delayed graft function (DGF), 16 of the 26 control patients were diagnosed with DGF (41.6 vs. 61.5%; *p* = 0.257). Average hospitalization did not significantly differ between patients in the ATLG group as compared with controls (AP-KTx: 16 days vs. CP-KTx: 12 days, *p* = 0.164; Table 1). Surgical, infectious or other complications did not significantly differ in the two study arms. The occurrence of adverse events is summarized in Table 2.

**Table 1:** Donor and recipient characteristics KTX.

**Variable**	**Total (*n* = 50)**	**ATG-perfused (*n* = 24)**	**Control-perfused (*n* = 26)**	***p*-value^#^**
Recipient age (yrs ± SD)^*^	54 ± 14	56 ± 13	53 ± 15	0.465
Recipient gender (females)^***^	14/50	7/24	7/26	1.000
Recipient BMI (kg/m2 ± SD)^*^	25 ± 5	25 ± 5	26 ± 5	0.571
Cold ischemic time (hours + IQR)^**^	13 (7)	11 (7)	14 (7)	0.222
Warm ischemic time (min + IQR)^**^	26 (10)	25 (10)	27 (8)	0.151
HLA Mismatch (number ± SD)^*^	3.4 ± 1	3.2 ± 2	3.6 ± 1	0.265
Panel reactive antibodies ≥20%^***^	0/50 (0%)	0/24 (0%)	0/26 (0%)	1.000
DISEASE^***^				
Polycystic kidney disease	11/50 (22%)	6/24 (25%)	5/26 (19%)	
Diabetes Typ II	5/50 (10%)	3/24 (13%)	2/26 (8%)	
IgA nephropathy	4/50 (8%)	1/24 (4%)	3/26 (12%)	
Glomerulonephritis	4/50 (8%)	2/24 (8%)	2/26 (8%)	
Hypertensive nephropathy	4/50 (8%)	2/24 (8%)	2/26 (8%)	
Intestinal or pyelonephritis	3/50 (6%)	2/24 (8%)	1/26 (4%)	
Alport syndrome	2/50 (4%)	0/24 (0%)	2/26 (8%)	
Renal vascular disease	1/50 (2%)	0/24 (0%)	1/26 (4%)	
Diabetes Typ I	1/50 (2%)	1/24 (4%)	0/26 (0%)	
Membranoproliferative glomerulonephritis	1/50 (2%)	0/24 (0%)	1/26 (4%)	
Chronic pyelonephritis	1/50 (2%)	0/24 (0%)	1/26 (4%)	
Amyloidosis	1/50 (2%)	1/24 (4%)	0/26 (0%)	
Focal segmental glomerulosclerosis	1/50 (2%)	1/24 (4%)	0/26 (0%)	
Analgesic nephropathy	1/50 (2%)	0/24 (0%)	1/26 (4%)	
Uncertain etiology	10/50 (20%)	5/24 (21%)	5/26 (19%)	
DGF (dialysis after Tx)	26/50	10/24	16/26	0.257
Recipient ICU stay (days + IQR)^**^	1 (1)	1 (1)	1 (2)	0.855
Hospital stay (days + IQR)^**^	13 (14)	16 (18)	12 (11)	0.164
Donor age (yrs ± SD)^*^	51 ± 17	51 ± 17	51 ± 18	0.933
Donor BMI (kg/m2 ± SD)^*^	26± 5	25 ± 4	26 ± 6	0.439
ECD Criteria^***^	21/50 (42%)	8/24 (33%)	13/26 (50%)	0.233

* Mean + standard deviation;

** median + interquartile range;

*** count (percentage);

# *differences between ATLG and control*.

**Table 2:** Adverse events KTX.

**Variable**	**Total (*n* = 50)**	**ATG-perfused (*n* = 24)**	**Control-perfused (*n* = 26)**
SURGICAL EVENTS^***^			
Ureter necrosis	3/50 (6%)	2/24 (8%)	1/26 (4%)
Vesicoureteral reflux	2/50 (4%)	0/24 (0%)	2/26 (8%)
Lymphocele	2/50 (4%)	0/24 (0%)	2/26 (8%)
Incisional hernia	2/50 (4%)	0/24 (0%)	2/26 (8%)
Bleeding	1/50 (2%)	1/24 (Tx loss)	0/26 (0%)
INFECTIONS^***^			
Polyomavirus	4/50 (8%)	2/24 (8%)	2/26 (8%)
CMV reactivation or infection	4/50 (8%)	2/24 (8%)	2/26 (8%)
Aspergillom	1/50 (2%)	0/24 (0%)	1/26 (4%)
Bacterial sepsis	1/50 (2%)	1/24 (4%)	0/26 (0%)
OTHERS^***^			
Histological-proven rejection	2/50 (4%)	1/24 (4%)	1/26 (4%)
Primary non-function	1/50 (2%)	0/24 (0%)	1/26 (4%)
Death (unknown reason)	1/50 (2%)	0/24 (0%)	1/26 (4%)
Prostate cancer	1/50 (2%)	1/24 (4%)	0/26 (0%)
Renal cell carcinoma (remnant kidneys)	1/50 (2%)	0/24 (0%)	1/26 (4%)

*** *Count (percentage) some patients had >1 event*.

### Renal function after kidney transplantation

**Figure 3:**
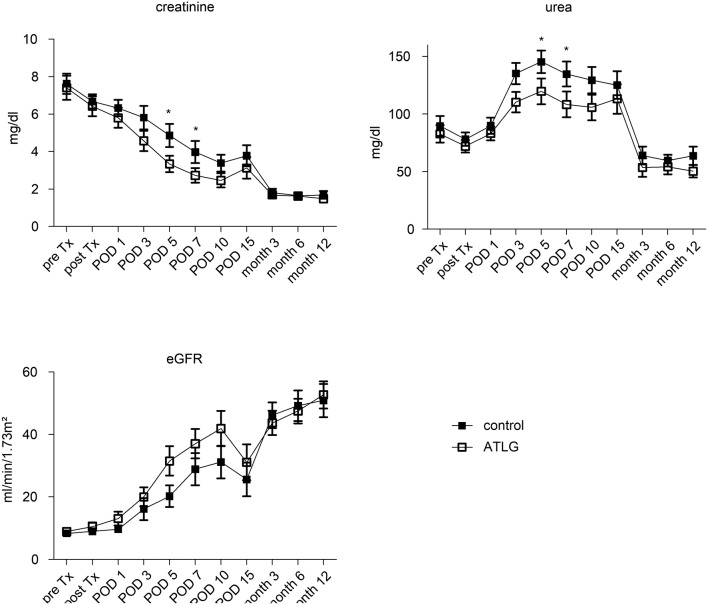
Clinical parameters following KTX. The functional parameters creatinine and urea as well as the calculated eGFR illustrate a comparable course between kidneys peri-operatively perfused with ATLG or saline eGFR until month 12 post-transplantation. However, ATLG recipients demonstrated significantly better graft function regarding creatinine as compared with controls at days 5 and 7 (*p* = 0.015 and *p* = 0.045). ATLG: open boxes, control: filled boxes; data are presented as mean values ± SEM; *p < 0.05.

Pre-transplantation, no differences in creatinine output were detected between the ATLG and the control group (AP-KTx: 7.4 ± 3.2 mg/dL vs. CP-KTx: 7.6 ± 2.7 mg/dL) (Figure 3), and creatinine levels steadily decreased in the early post-transplantation period until day 10. With respect to our defined primary endpoint, ATLG recipients demonstrated significantly better graft function regarding creatinine as compared with controls at day 7 (AP-KTx: 2.7 mg/dL ± 1.9 mg/dL vs. CP-KTx: 4.0 mg/dL ± 2.9 mg/dL; *p* = 0.045) and even at day 5 (AP-KTx: 3.3 mg/dL ± 2.2 mg/dL vs. CP-KTx: 4.8 mg/dL ± 3.1 mg/dL, *p* = 0.015). Similar observations were made for urea, illustrating significantly lower values at days 5 and 7 for the ATLG group as compared with the controls (day 5: AP-KTx: 119.8 mg/dL ± 54.8 mg/dL vs. CP-KTx: 145.4 mg/dL ± 48.7 mg/dL, *p* = 0.038 and day 7: AP-KTx: 108.3 mg/dL ± 53.7 mg/dL vs. CP-KTx: 134.8 mg/dL ± 53.6 mg/dL; *p* = 0.031). From day 15 until 3 months post transplantation creatinine and urea showed a clear decline and remained stable for the observation period of 12 months, illustrating no further differences between ATLG and control patients. Although the calculated estimated glomerular filtration rate (eGFR) revealed better graft function for patients receiving ATLG-perfused kidneys in the early transplantation phase, these differences were not statistically relevant (Figure 3).

### Peri-operative perfusion of the kidney with ATLG does not result in inflammatory changes

**Figure 4:**
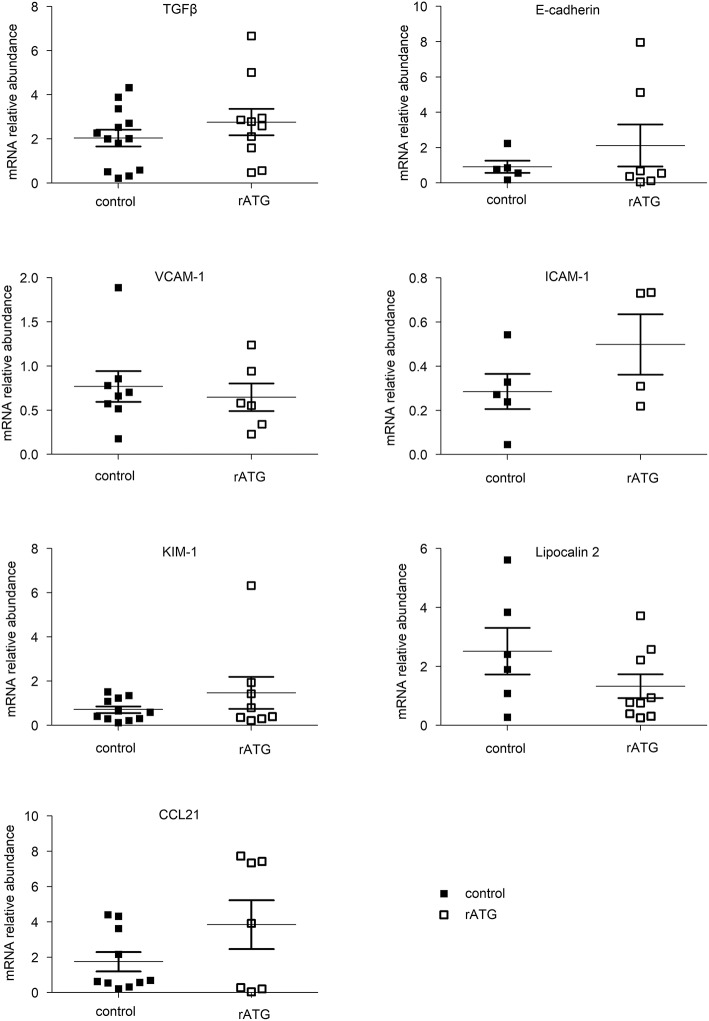
mRNA analysis of candidate genes in kidney biopsies taken 1 h post-reperfusion. Data from available tissue specimens are shown. mRNA measurement of selected candidate genes indicative for inflammation and adhesion did not reveal significant differences between ATLG-perfused kidneys and control kidneys. ATLG: open boxes, control: filled boxes. Data are presented as mean ± SEM.

In order to evaluate potential effects on intragraft inflammatory expression pattern, we analyzed renal biopsies retrieved 1 h post-reperfusion of the two kidney cohorts. From the available biopsy specimens we analyzed adhesion molecules including *intercellular adhesion molecule 1* (ICAM-1), *vascular cell adhesion molecule 1* (VCAM-1) and E-Cadherin by gene expression analysis, in order to study potential effects of ATLG perfusion. However, no differences were detected between ATLG- and control-perfused kidneys for these markers. Moreover, additional candidate genes being indicative for kidney injury including *transforming growth factor beta* (TGFß), the *kidney injury molecule 1* (KIM-1) or Lipocalin 2 (LCN2) further did not show any significant differences between the investigated groups. To examine whether genes responsible for cell migration are affected by ATLG perfusion, we exemplarily investigated the CC-chemokine ligand 21 (CCL21), which has been shown by us and others to be upregulated during renal rejection (26, 27). Again, no significant differences for CCL21 gene expression could be observed (Figure 4).

### Impact of kidney transplantation on peripheral blood lymphocytes post-transplantation

**Figure 5:**
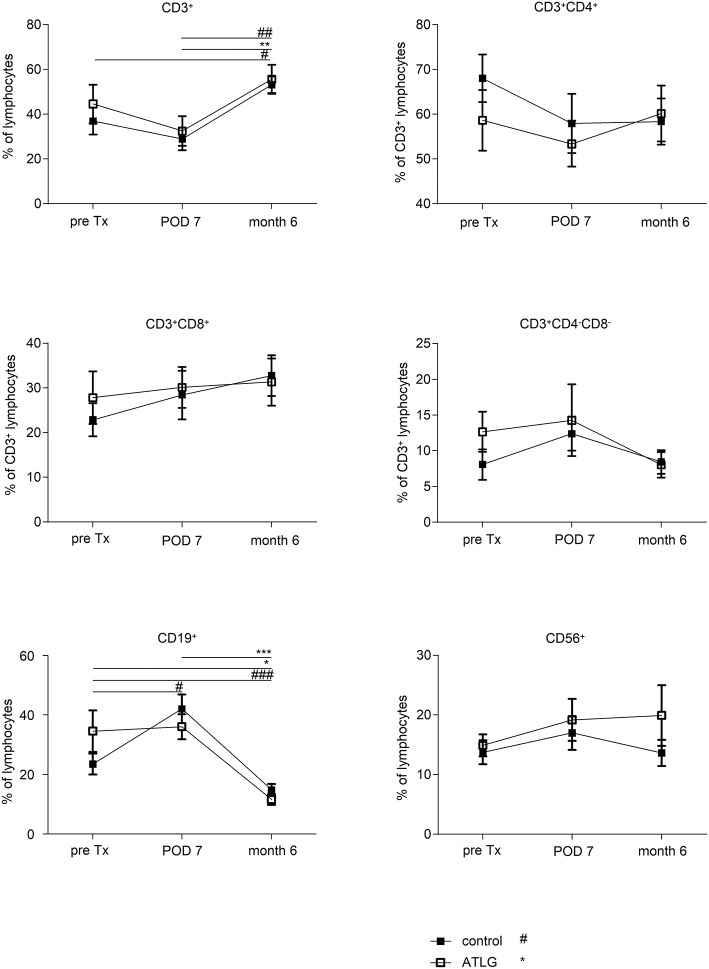
Frequencies of major lymphocyte subsets in the peripheral blood of kidney recipients. Recipients who received an ATLG-perfused organ and those who received a saline-perfused organ illustrated similar frequencies of CD3^+^, CD4^+^, CD8^+^ T cells, CD4^−^CD8^−^ T cells, CD19^+^ B cells, and CD56^+^CD3^−^ NK cells. Interestingly, whereas T cell frequencies appeared to decrease in the early postoperative days, there was a significant increase between day 7 post-transplantation (POD 7) and month 6 (*p* = 0.0078, respectively). Moreover, both patient groups show a significant decrease in CD19^+^ B cells as compared with pre-transplant levels and POD7 (*p* = 0.001, respectively). ATLG: open boxes, control: filled boxes; data are presented as mean ± SEM; *p < 0.05, **p ≤ 0.01, ***p ≤ 0.001; *, difference within AP; #, difference within CP; ^*##*^ < 0.01, ^*###*^ < 0.001.

We further investigated various lymphocyte subsets in the peripheral blood of kidney recipients. In general, no fundamental differences between the intervention and the control group were detected, but we observed clear transplant-related changes for various blood lymphocyte subsets. For instance, CD3^+^ T cell frequencies declined during the early transplantation period for both groups (until day 7 post-tx), finally resulting in a significant increase by month 6 (AP-KTx: 32.5% ± 18.7% vs. 55.7% ± 18.4%, *p* = 0.0078; CP-KTx: 28.9 ± 14.4 vs. 53.2 ± 10.6, *p* = 0.0078; day 7 vs. month 6, respectively). Decreased frequencies during the early post-transplant period appear to be more evident for CD4^+^ T cells, although not statistically significant (Figure 5). Interestingly, the most dramatic change was observed for CD19^+^ B cells. Whereas B cells remained unaffected in the ATLG group until day 7 post-transplantation, their frequencies significantly increased until day 7 in the control group (CP-KTx: 23.55% ± 12.8% vs. 42.1% ± 15.9%, pre Tx vs. day 7; *p* = 0.042). However, compared with pre-transplant levels and day 7 both kidney recipient groups exhibited a significant decrease in CD19^+^ B cells at month 6 (AP-KTx: 36.1% ± 15.8% vs. 11.6% ± 5.8%, *p* = 0.001; CP-KTx: 43.1% ± 15.9% vs. 14.9% ± 7.5%, *p* = 0.001; day 7 vs. month 6, respectively). No further significant changes were detected for CD8^+^ T cells, double-negative (DN) CD4^−^CD8^−^ T cells, or CD56^+^CD3^−^ NK cells (Figure 5). Focusing on T cell subsets, ATLG resulted in induction of naïve CD4^+^ and naïve CD8^+^ T cells as well as in CD4^+^ and CD8^+^ central memory T (T_CM_) cells in the early post-transplantation period, although this observation was only significant for CD4^+^ T_CM_ (AP-KTx: 7.3% ± 6.7% vs. 19.46% ± 16.4%, *p* = 0.006; pre Tx vs. day 7) (Supplemental Figure 2).

### Age-related effects of ATLG perfusion

**Figure 6:**
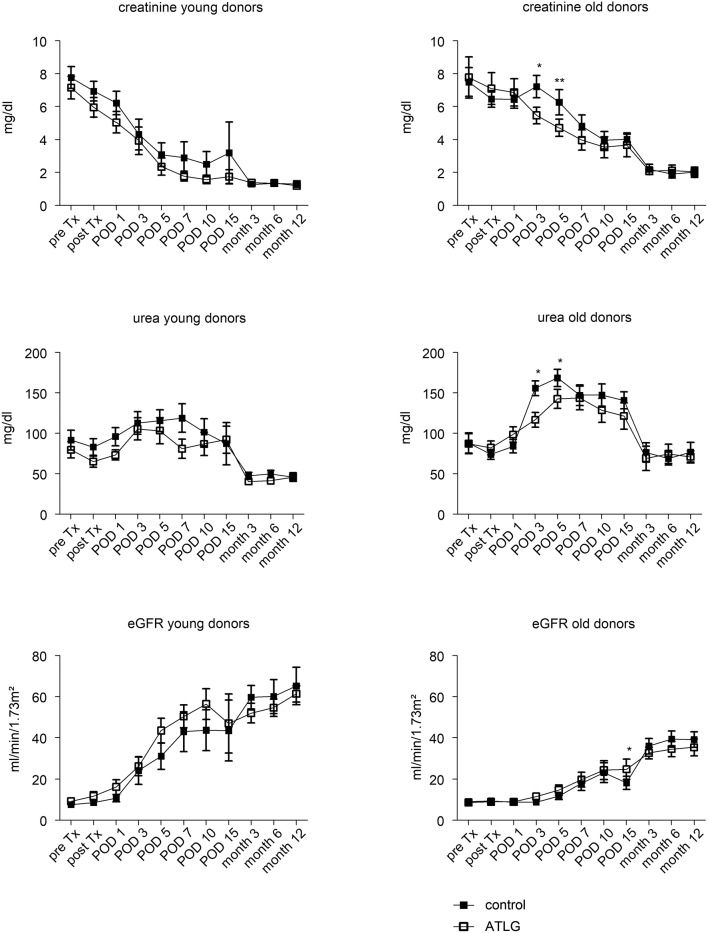
Age-related effects of ATLG perfusion. Creatinine, urea levels and the calculated eGFR are displayed for donor organs grouped into either young (≤55 years, *n* = 14 for rATG perfused) or old organs (>55 years, *n* = 10, rATG perfused). Accordingly, donor organs were also grouped in young (≤55 years, *n* = 12) or old (>55 years, *n* = 14). ATLG: open boxes, control: filled boxes; data are presented as mean values ± SEM; *p < 0.05, **p ≤ 0.01.

**Figure 7:**
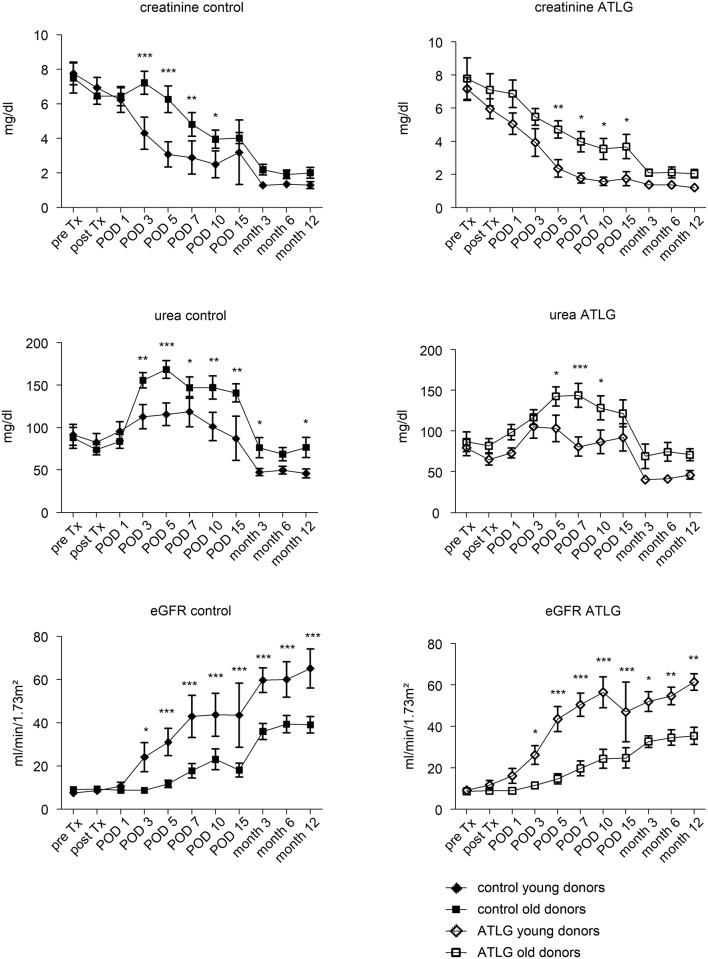
Age-related effects of young and old donor organs. Within the rATG and control group, graft outcome was separately analyzed for young (≤55 years) or old (>55 years) donor age. ATLG: open boxes, control: filled boxes; data are presented as mean values ± SEM; *p < 0.05, **p ≤ 0.01, ***p ≤ 0.001.

We recently addressed whether 0 h biopsies reveal markers for cellular senescence, illustrating that the majority of candidate genes were upregulated in donor organs aged >55 years (28). In order to evaluate potential age-related effects of ATLG perfusion, we chose an analogous cut-off to dichotomize donor groups in ATLG ≤55 years (APD young; *n* = 14, donor mean age 38.9) and >55 years (APD old; *n* = 10, donor mean age 66.9) as well as for the control group ≤55 years (CPD young; *n* = 12 mean age 35.6) and >55 years (CPD old; *n* = 14 mean age 64.2). Whereas no differences were detectable between APD young and CPD young organs, we observed significantly better graft function in the APD old group as compared to the CPD old group. This was reflected by creatinine and urea values at day 3 and day 5 post-transplantation (creatinine: *p* = 0.015 and *p* = 0.008; urea: *p* = 0.013; *p* = 0.047, Figure 6). However, when comparing aged vs. young donor organs within the groups, indeed the younger organs display significantly better graft function than do the aged organs within the ATLG and the control group. This observation remained significant until the end of the observation period of 12 months (Figure 7).

### Patient and graft survival rates 1 year post-transplantation

**Figure 8:**
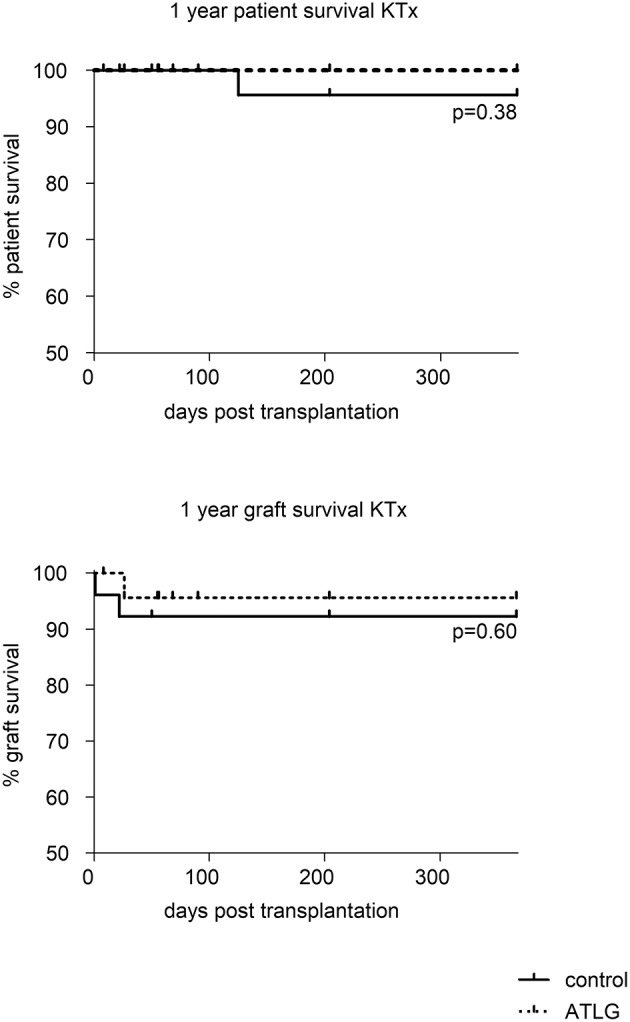
Patient and graft survival at 1 year post-transplantation. Whereas no differences in patient as well as graft survival were detected between ATLG-perfused kidneys and control kidneys. (patient survival: AP-KTx 100% vs. CP-KTx 96%, *p* = 0.38; graft survival: AP-KTx 96% vs. CP-KTx 92%, *p* = 0.60).

Although primary the endpoint displayed a significant difference for functional short term outcome in the treatment group, no differences in patient nor graft survival were observed at the end of the observation period (patient survival: AP-KTx 100% vs. CP-KTx 96%, *p* = 0.38; graft survival: AP-KTx 96% vs. CP-KTx 92%, *p* = 0.60). Organ losses were due to bleeding (AP-KTx group) and ureter necrosis and primary non-function (PNF) (CP-KTx group). Death occurred in only one patient in the control group for unknown reason. Kaplan-Meier plots for graft survival demonstrate a similar trend (Figure 8).

## Discussion

The conditioning of ECD or DCD transplants in order to optimize graft quality for better transplant outcome currently represents an urgent need in transplantation medicine. Whereas the treatment of brain dead donors has been addressed in only a small number of clinical trials (29–31), conditioning of the organ by machine perfusion is currently considered in a clearly larger number of clinical trials (4, 6, 32–34). These studies mainly address the impact of hypothermic vs. cold storage machine perfusion, whereas alternative approaches for organ conditioning are tested solely in pre-clinical models (35–37) or have been proven insufficient (38). In this context, targeting IRI by applying various treatment schemes has been illustrated in an extensive number of experimental trials (37, 39, 40). For instance, ATLG was seen to be effective in preventing IRI-associated renal function impairment, tissue damage and tubular apoptosis when applying ATLG to the recipient (41). This effect seemed to be related to the various antibodies present in anti-thymocyte preparations affecting the binding and surface expression of integrins (LFA-1, VLA-4) and adhesion molecules involved in leukocyte/endothelial cell interaction (42). Moreover, in a warm ischemia kidney model, application of rATG resulted in reduced cell necrosis, cytoplasmic vacuolization, cast formation, and tubular dilatation among ATG-treated rats (43). As ATLG is an established immunosuppressant in SOT, we aimed to address whether peri-operative perfusion with ATLG results in amelioration of IRI by targeting adhesion- and inflammation-induced molecules in a kidney transplantation setting. To the best of our knowledge, this is the first clinical trial that aims to optimize solid organs by perfusion with antibodies.

In KTx, we detected improved renal function in the early post-operative days as evidenced by lower creatinine and urea levels at days 5 and 7 in the ATLG Group (Figure 3) that meet the defined clinical primary endpoint. The beneficial effect of ATLG was also demonstrated by the lower incidence of DGF in the ATLG Group (Table 1). In an explorative evaluation, it appeared that this effect was even more pronounced in older organs (Figure 6). However, we were not able to detect differences in graft function between ATLG and control patients at the end of the 12-month observation period.

Gene expression profiling of the organs did not reveal differences in markers indicative for inflammation and adhesion in kidney transplantation setting. In addition, no differences were obtained for the main investigated lymphocyte subsets with the exception of B cells, which demonstrated a significant decrease at month 6 as compared to pre-transplant frequencies in both kidney recipient groups. This shows that this effect was unrelated to perfusion with ATLG (Figure 5) confirming previous observations that B cells decline post kidney transplantation, but illustrating a higher differentiation status (44). The reason for this phenomenon remains to be clarified.

Based on our observations, two pressing questions arise. First, why ATLG-perfused kidneys show better graft function, although we could not detect an influence of ATLG perfusion in the allografted tissue at the molecular level. Second, why older donor organs should benefit to a greater extent from ATLG conditioning than younger organs although we were not able to detect even subtle changes in the allograft. With respect to the first question, we can only speculate that the time between peri-operative perfusion of the organ and withdrawal of the renal biopsy 1 h post-reperfusion was too short for detection of mRNA changes. However, the benefit of ATLG in the KTx setting is obvious and suggests that when binding to endothelial antigens happened, adhesion, rolling and migration of leukocytes into the tissue appeared to be ameliorated. As the limited amount of material prevented us from evaluating the degree of tissue infiltration, this assumption indeed remains speculative and further studies are needed to address the potential effect of ATLG perfusion *in situ*. Regarding the second question, the general paradigm has been generated that aged organs display greater immunogenicity than do younger organs by containing larger numbers of dendritic cells that illustrate enhanced MHC II expression, thus provoking a more intense alloimmune response (45, 46). We therefore hypothesize that aged organs exhibit stronger expression of endothelial antigens and therefore benefit to a greater extent from ATLG perfusion than do younger grafts.

We are aware of several limitations of our study. First, the study's relatively small sample size, which likely contributed to underpowering to meet the defined study secondary endpoints. Second, the duration of organ perfusion and incubation with ATLG solution for 5 min. might not be ideal for effective binding of the polyclonal antibody to potential antigens. Upcoming studies will clarify whether prolonged incubation with ATLG or a combination with machine perfusion will contribute to a more pronounced effect. However, perfusing solid organs with ATLG might appear safer than the application of ATLG as induction therapy shortly after surgery, as we demonstrated stable lymphocyte populations post-transplantation. Recent data from a large study cohort suggest that induction therapy with rATG results in serum sickness disease, which is a risk factor for graft loss in the long-term (47). Moreover, rATG has been identified as being associated with a greater risk for viral and bacterial infections post-transplantation (17).

In conclusion, our data provide robust support for new concepts in the peri-operative conditioning of solid organs. First, the combination of machine perfusion and additional agents including antibodies or alternative therapeutics (48, 49) may provide a synergistic effect with regard to organ preservation and reduction of IRI. Second, consideration of the influence of age is strongly recommended for upcoming clinical trials.

## Author contributions

PR, JP, and KK substantially contributed to the conception and design of the study. PR, JG, LH, StE, SuE, AK, AS, FF, SW, CB, AW, RO, BC, RÖ, SS, MB, CD, CM, TR, FA, and MM supported acquisition of data, or analysis and interpretation of data. PR and KK were responsible for writing the manuscript. All authors revised the final version approved publication.

### Conflict of interest statement

The authors declare that the research was conducted in the absence of any commercial or financial relationships that could be construed as a potential conflict of interest.
