# Supplementary material for: Graft Pre-conditioning by Peri-Operative Perfusion of Kidney Allografts With Rabbit Anti-human T-lymphocyte Globulin Results in Improved Kidney Graft Function in the Early Post-transplantation Period—a Prospective, Randomized Placebo-Controlled Trial
Source: Front Immunol. 2018 Aug 24;9:1911. doi: 10.3389/fimmu.2018.01911 (PMC6117415; doi:10.3389/fimmu.2018.01911)
Supplement: Supplementary file 1 [file Table_1.DOCX]

**Supplemental Table 1. Antibodies/Clones applied for FACS staining**

| **Antibody** | **Clone** | **Isotype** | **Dilution** | **Conjugate** | **Company** | **Catalog Nr.** |
| --- | --- | --- | --- | --- | --- | --- |
| CD3 | HIT3a | Mouse IgG_2a_, κ | 1:100 | BV 510 | BD Biosciences | 564713 |
| CD4 | SK3 | Mouse IgG_1_, κ | 1:100 | BV 786 | BD Biosciences | 563877 |
| CD4 | RPA-T4 | Mouse IgG_1_, κ | 1:100 | PE-CF594 | BD Biosciences | 562281 |
| CD8 | RPA-T8 | Mouse IgG_1_, κ | 1:20 | AF 700 | BD Biosciences | 557945 |
| CD8 | Hit8a | Mouse IgG_1_, κ | 1:20 | FITC | BD Biosciences | 555634 |
| CD14 | MyP9 | Mouse IgG_2b_, κ | 1:100 | BV 711 | BD Biosciences | 563372 |
| CD16 | 3G8 | Mouse IgG_1_, κ | 1:20 | PE | BD Biosciences | 555407 |
| CD19 | HIB19 | Mouse IgG_1_, κ | 1:10 | PE-Cy7 | ebiosciences | 25-0199-42 |
| CD45 | 2D1 | Mouse IgG_1_, κ | 1:100 | APC-H7 | BD Biosciences | 560178 |
| CD45 RA | HI100 | Mouse IgG_2b_, κ | 1:100 | BB515 | BD Biosciences | 564552 |
| CD56 | AF12-7H3 | Mouse IgG_1_, κ | 1:50 | APC | Miltenyi | 130-090-843 |
| CD62L | DREG-56 | Mouse IgG_1_, κ | 1:100 | BV 650 | BD Biosciences | 563808 |
| CD64 | 10.1 | Mouse IgG_1_, κ | 1:100 | BV 421 | BD Biosciences | 562872 |
| 7AAD |  |  | 1:50 | 7AAD | BD Biosciences | 559925 |
