# Supplementary material for: Graft Pre-conditioning by Peri-Operative Perfusion of Kidney Allografts With Rabbit Anti-human T-lymphocyte Globulin Results in Improved Kidney Graft Function in the Early Post-transplantation Period—a Prospective, Randomized Placebo-Controlled Trial
Source: Front Immunol. 2018 Aug 24;9:1911. doi: 10.3389/fimmu.2018.01911 (PMC6117415; doi:10.3389/fimmu.2018.01911)
Supplement: Supplementary file 2 [file Table_2.DOCX]

**Supplemental Table 2. Primer sequences used for RT-PCR**

| **Gene** | **Company** | **Catalog Nr.** |
| --- | --- | --- |
| TGFß | Life Technologies | Hs00998133_m1 |
| E-cadherin | Life Technologies | Hs01023895_m1 |
| VCAM-1 | Life Technologies | Hs01003372_m1 |
| ICAM-1 | Life Technologies | Hs00164932_m1 |
| KIM-1 | Life Technologies | Hs00930379_g1 |
| Lipocalin 2 | Life Technologies | Hs01008571_m1 |
| CCL21 | Life Technologies | Hs00171076_m1 |
