# Supplementary figures and images for: Graft Pre-conditioning by Peri-Operative Perfusion of Kidney Allografts With Rabbit Anti-human T-lymphocyte Globulin Results in Improved Kidney Graft Function in the Early Post-transplantation Period—a Prospective, Randomized Placebo-Controlled Trial
Source: Front Immunol. 2018 Aug 24;9:1911. doi: 10.3389/fimmu.2018.01911 (PMC6117415; doi:10.3389/fimmu.2018.01911)

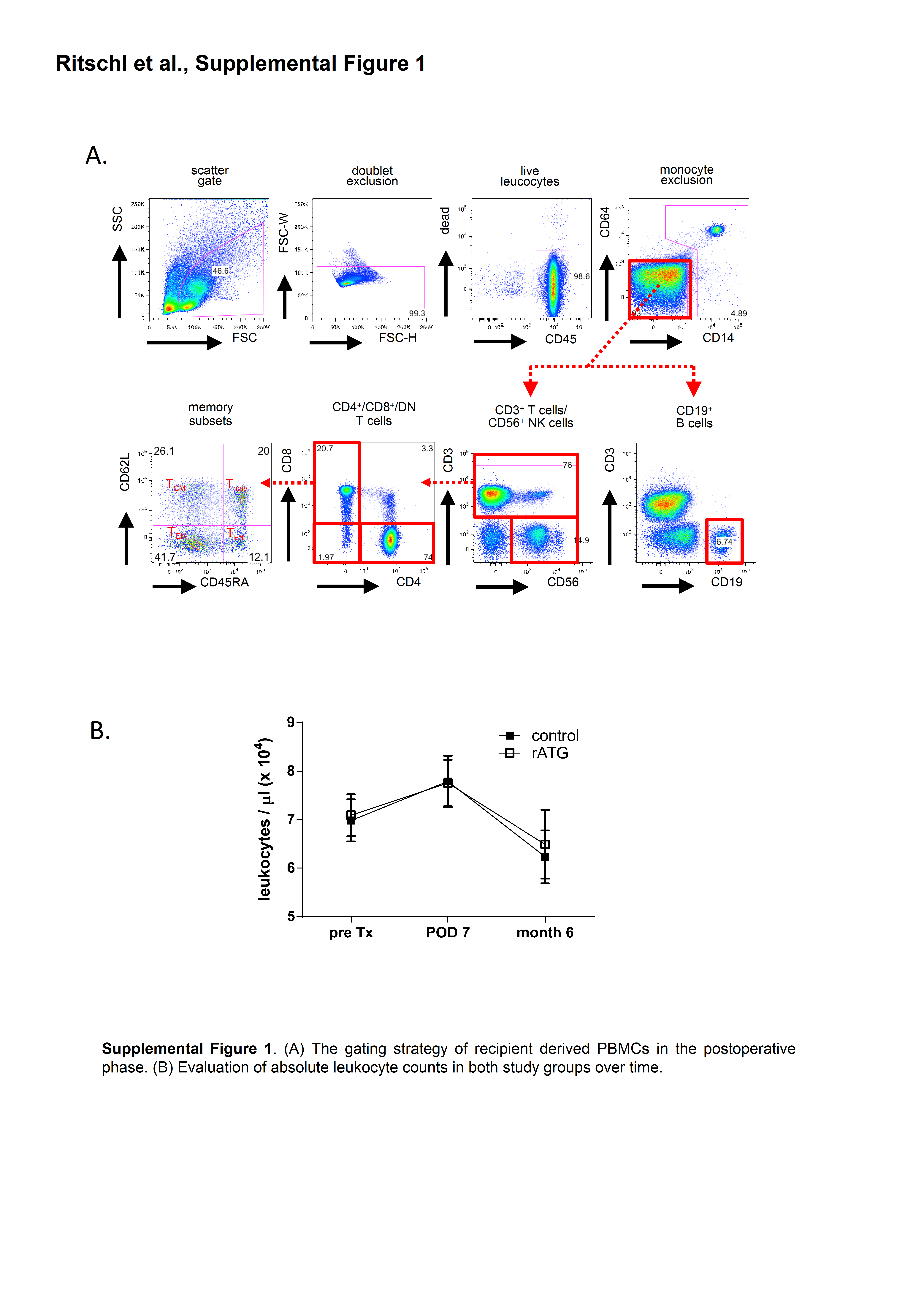

Supplement: Supplementary file 3 [file Image_1.TIF]

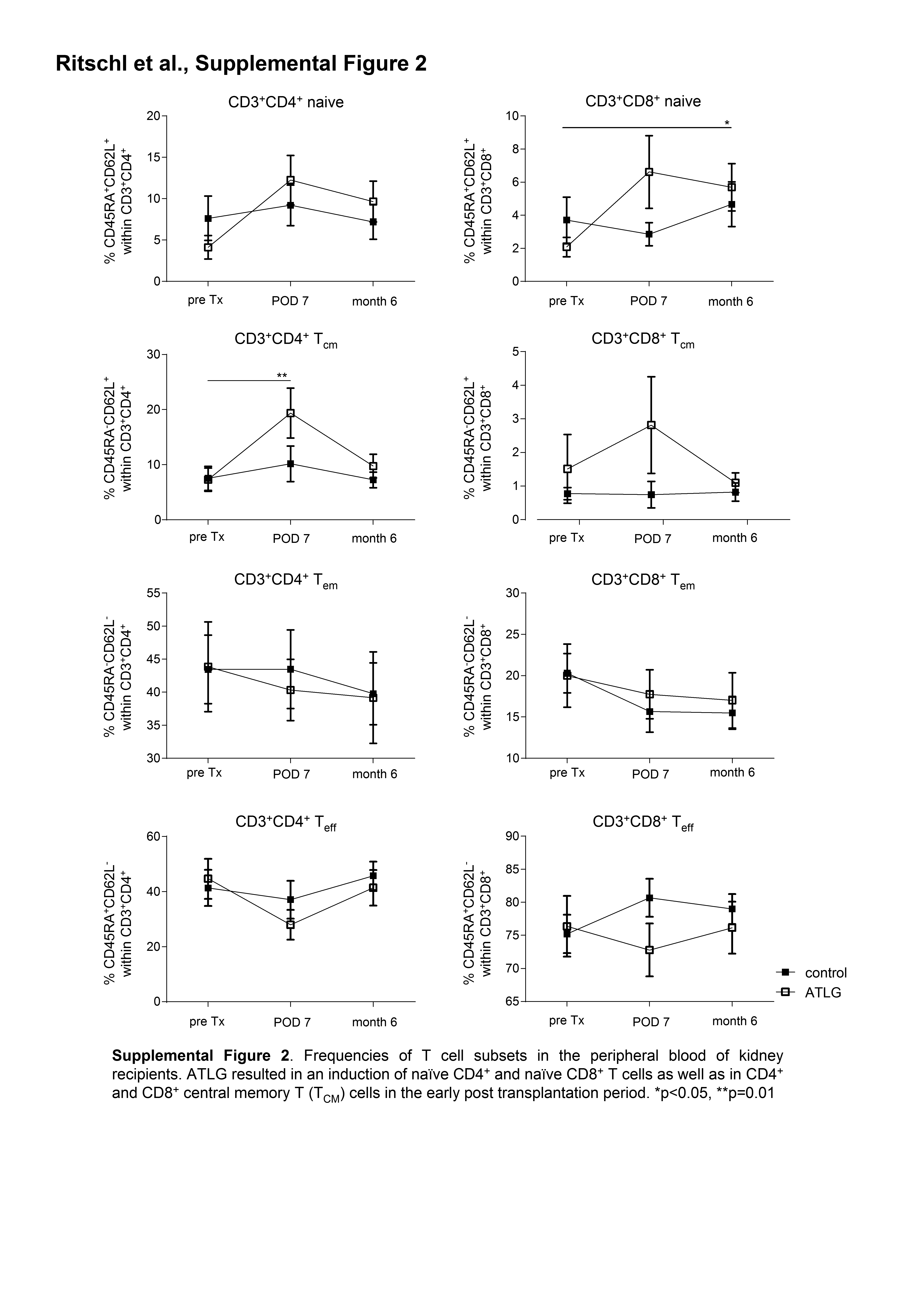

Supplement: Supplementary file 4 [file Image_2.TIF]
